# Identification of novel candidate genes associated with non-syndromic tooth agenesis in Mongolian families

**DOI:** 10.1007/s00784-023-05415-2

**Published:** 2023-12-29

**Authors:** Dejidnorov Semjid, Hyunsoo Ahn, Sapaar Bayarmagnai, Munkhjargal Gantumur, Sanguk Kim, Jae Hoon Lee

**Affiliations:** 1 Department of Prosthodontics, College of Dentistry at Yonsei University, 50-1 Yonsei-Ro, Seodaemoon-Gu, Seoul, 120-752 Republic of Korea; 2 Department of Life Sciences, Pohang University of Science and Technology, 80 Jigok-Ro, Nam-Gu, Pohang, 790-784 Republic of Korea; 3 Department of Prosthodontics, School of Dentistry, Mongolian National University of Medical Sciences, Chingeltei District, Nuuriin 2-21, Ulaanbaatar, Mongolia

**Keywords:** Tooth agenesis, Genetic variants, Whole-exome sequencing, Bioinformatic analysis, In silico mutation, Mongolian population

## Abstract

**Objectives:**

This study aimed to identify genetic variants associated with non-syndromic tooth agenesis (TA) in nine families from Mongolia using whole-exome sequencing (WES) and bioinformatics analysis.

**Material and methods:**

The study enrolled 41 participants, including three inherited and six non-inherited families. WES analysis was performed on 14 saliva samples from individuals with non-syndromic TA. The potential candidate genes were identified through variant filtering and segregation analysis. The filtered variants were then analyzed in silico mutation impact analysis.

**Results:**

WES analysis identified 21 variants associated with TA, and 5 of these variants met all filtering criteria. These variants were located in the exome region of MAST4, ITGA6, PITX2, CACNA1S, and CDON genes. The variant in PITX2 was found in eight participants from inherited and non-inherited families, while the MAST4 variant was identified in 6 participants from inherited families.

**Conclusions:**

The study identified various genetic variant candidates associated with TA in different family groups, with PITX2 being the most commonly identified. Our findings suggest that MAST4 may also be a novel candidate gene for TA due to its association with the Wnt signaling pathway. Additionally, we found that five candidate genes related to focal adhesion and calcium channel complex were significant and essential in tooth development.

**Clinical relevance:**

Identifying new pathogenic genes associated with TA can improve our understanding of the molecular mechanisms underlying the disease, leading to better diagnosis, prevention, and treatment. Early detection of TA based on biomarkers can improve dental management and facilitate orthodontic and prosthetic treatment.

**Supplementary Information:**

The online version contains supplementary material available at 10.1007/s00784-023-05415-2.

## Introduction

TA is a common developmental anomaly that results in the congenital absence of one or more permanent teeth in humans. It affects approximately 200 million individuals worldwide, and its incidence varies by geography, population, and race [1, 2]. TA can lead to several complications, including oral symptoms, masticatory dysfunction, physiological issues, speech impairments, aesthetic concerns, and financial burdens, which can significantly affect the quality of life [3, 4]. Tooth development begins with sequential and reciprocal interactions between embryonic tissues, such as oral epithelium and ectomesenchyme [5]. This process is regulated by various signaling pathways, including BMP, FGF, SHH, TNF, and WNT, which are essential for the tooth bud, cap, and bell stages [6, 7]. Disruptions in these signaling pathways during tooth development may cause TA [8].

TA can be classified into non-syndromic or syndromic based on the involvement of other organs or tissues. Non-syndromic TA, also known as selective TA, is characterized by dental abnormalities without other tissue symptoms. On the other hand, syndromic TA involves missing teeth and other developmental abnormalities such as nails, hair, or skin. Some examples of syndromic TA include cleft lip/plate [9], ectodermal dysplasia [10], Axenfeld-Rieger syndrome, and Witkop syndrome [11–13]. The etiology of TA can be attributed to genetic or environmental factors [14–17]. However, genetic factors play a more significant role in the pathogenesis of TA [18, 19]. Identifying the genetic factors associated with TA can improve diagnosis and treatment. Several genes have been identified, including MSX1 [20], PAX9 [14], BMP4 [21], AXIN2 [22], EDA [23], EDAR [24], EDARADD [25], WNT10A [4], WNT10B [26], LRP6 [27], PITX2 [28], FGFR2 [3], and CACNA1S [3]. These genes exhibit autosomal-dominant, autosomal-recessive, and X-linked mechanisms of inheritance [29]. MSX1 and PAX9 are among the first genes associated with TA, and their protein products act as transcription factors essential for the tooth germ’s development from mesenchymal cells [30, 31]. Mutations in AXIN2 and WNT10A can disrupt the Wnt signaling pathway, which is vital for tooth development, with AXIN2 involved in tooth germ formation and WNT10A in the differentiation of dental mesenchymal cells [15]. Recently, it has been shown that PITX2 contributes to TA and is involved in the early stages of tooth development, including the formation of tooth germs and bud morphology [32].

In recent years, the next-generation sequencing (NGS) approach, specifically WES platforms, such as Illumina, has been extensively used for identifying biomarkers of genetic disease diagnosis [33]. Although only 2% of the human genome consists of exons, 85% of the genetic variations responsible for highly penetrant diseases reside in this small genome region [28]. WES has improved diagnostic accuracy, shortened the diagnostic process, and is more cost-effective than traditional methods. This approach identifies genetic variations that may contribute to the development of TA. Bioinformatic analysis can then be used to analyze the WES data [34]. It can help identify potential candidate genes and variants that may contribute to the condition, especially in a limited number of patients. Furthermore, it can help predict the functional impact of genetic variants and provide insights into the pathogenesis of the condition.

Our study aims to investigate the genetic variants of non-syndromic TA in Mongolian families by utilizing WES and bioinformatic analysis.

## Materials and methods

### Subjects

A total of 41 individuals from nine Mongolian families were enrolled in the study. Among these individuals, 15 were identified with non-syndromic TA. However, in Family 1, one participant (I-2) who had non-syndromic TA was unable to provide saliva samples for analysis. Consequently, 14 participants who had both clinical data and saliva samples available underwent WES analysis. Participants with TA were identified through clinical examination and panoramic radiographs. It was confirmed that the missing permanent teeth were not due to extraction or injuries. The general clinical examination confirmed that all study participants exhibited non-syndromic TA and had normal hair, skin, sweat glands, facial features, and nails.

### Radiographic assessment

Panoramic radiographs were obtained from 14 participants.

### Sample collection

Saliva samples were collected from each participant using the Oragene DNA Self-Collection Kit manufactured by DNA Genotek Inc., located in Ottawa, Canada. A 2 ml saliva was then collected and subsequently mixed with a DNA-preserving solution in a tube, following the guidelines set forth by the manufacturer. These collected DNA samples were then sent to DNA Link Inc., located in Seoul, South Korea, for further analysis, including extraction and additional testing.

### Control group

The study utilized exome sequencing data from a randomized subsample of 100 healthy individuals (Koreans), obtained from the Ansan-Ansung population consisting of 3703 individuals. These subsamples were provided by the Korea BioBank, Center for Genome Science, National Institute of Health, Korea Centers for Disease Control and Prevention. The individuals selected from the reference population were chosen randomly, regardless of sex and age, and had no history of significant diseases.

### Whole-exome sequencing

DNA samples were processed for WES using the SureSelectXT Human All Exon V5 kit and Novaseq6000 platform. The DNA quality was assessed through 1% agarose gel electrophoresis and PicoGreen® dsDNA Assay. The library was prepared by fragmenting genomic DNA, ligating sequencing adapters, and amplifying the adapter-ligated DNA using PCR. A hybridization buffer was then prepared by mixing SureSelect hyb #1, #2, #3, and #4 reagents, and the amplified DNA fragments were concentrated and SureSelect blocks #1, #2, and #3 reagents were added. The DNA-blocking agent mixture and hybridization buffer were incubated, and a RNase block was added to the SureSelect oligo capture library, which was then incubated. After adding the hybridization buffer and DNA blocking agent mix to the capture library, the mixture was incubated at 65 $^\circ{\rm C}$ for 24 h. The captured library was then washed with SureSelect binding buffer and eluted with nuclease-free water. Finally, the library was amplified and tagged with index tags. The libraries were pooled in equimolar amounts and subjected to sequencing using the Illumina Novaseq 6000 system following the protocol for 2 × 100 sequencing.

### Family-level statistical analysis of WES data and variant filtering

The study grouped patients from the same family into one subgroup to enhance the effects of a family inheritance. If not, each family’s sole patients are placed in the opposite grouping. The WES data were used to identify variants, with only protein-coding transcripts being taken into account.

Fisher’s exact test was used to compare the allele frequencies of each mutation between the control and each subgroup. To qualify risky variants of TA, variants with association *p* < 0.05 and ORs > 1 were considered statistically significant (Supplement Table 3). The threshold of Odds ratio (OR) was set as > 1, which just implies that the variant ratio was higher in the patient subgroup than in the control cohort, Then, the significant associations of the variant were evaluated with *p*-values. After statistical analysis, the study subsequently collected variants that can be associated with TA by disrupting protein function. To reduce the systematic differences between the sample subgroups, we filtered variants on TA-related genes with public databases of gene-phenotype relationships. This step can limit the range of variants to our interest, which can reduce the noise signal from the other variants. Variants with annotation of HIGH impact in SnpEff [35] and variants with MODERATE impact (Supplement Table 1) but are predicted to damage its function with the results of computational prediction tools (PolyPhen2 [36] and SIFT [37]) or lower minor allele frequency in the Asian population (ASN MAF [38]) than 0.1 were filtered. Next, the study filtered variants related to TA with associated gene lists from Open Targets (OT) [39] (EFO_0005410, tooth agenesis), Gene Ontology (GO) [40] (GO:0042476, odontogenesis), and Human Phenotype Ontology (HPO) [41] (HP:0000677, oligodontia).

### In silico mutation analysis

Sorting Intolerant From Tolerant (SIFT) [37] and Polymorphism Phenotyping v2 (PolyPhen2) [36] to detect mutations that may be responsible for TA. All possible non-redundant protein sequences in Ensembl database were analyzed for the two missense variants. SIFT determines the sequence conservation across multiple species, assuming mutations in highly conserved regions to be intolerable. PolyPhen2 predicts mutational impact by utilizing sequence co-evolution and protein structure. Table 3 summarizes the candidate genes with identified variants and their mutation impact analysis.

### Gene set enrichment analysis

The g: Profiler [42] was used to perform gene set enrichment analysis (GSEA) for the candidate genes. The functional terms were derived from various data sources, including Gene Ontology [43] (GO molecular function, GO cellular component, and GO biological process), as well as biological pathways such as KEGG [44] and Reactome [45]). To increase the functional association of each gene, the gene sets were expanded by adding neighbor genes in the protein–protein interaction (PPI) network. The STRING (v11.5) [46] database was used for this process, with a confidence level of 700. The resulting network showed the relationship among the expanded gene sets, while the plot displays the function terms significantly enriched in the candidate genes at p_adj_ < 0.01.

## Results

### Study subjects

**Fig. 1:**
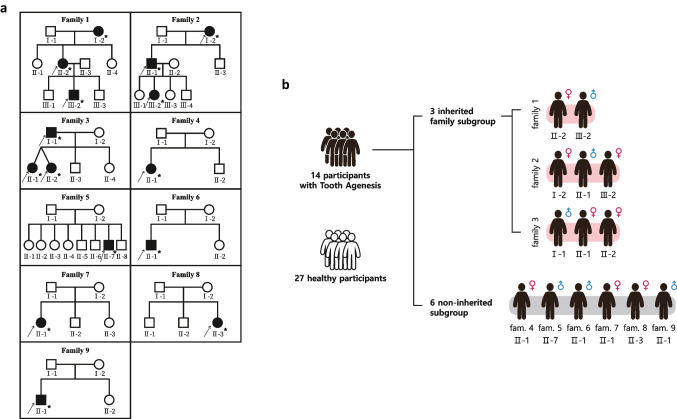
Pedigrees of the nine families and subgroup assignment of family members in the study. **a** Squares indicate males, and circles indicate females. Filled symbols represent individuals diagnosed with non-syndromic TA, while empty circles indicate unaffected subjects. Arrows indicate the probands of each family. The asterisks represent subjects who underwent WES analysis. **b** Classification assignment of family members in subgroups

The present study included nine Mongolian families, three of which were inherited families, as shown in Fig. 1a. We performed WES analysis on a total of 14 samples, consisting of eight females and six males, with ages ranging from 8 to 68 years (Fig. 1b and Supplement Table 4). All 14 individuals were clinically diagnosed with non-syndromic TA, which was further confirmed through intra-oral examination and panoramic radiograph.

### Clinical findings of families with non-syndromic TA

**Fig. 2:**
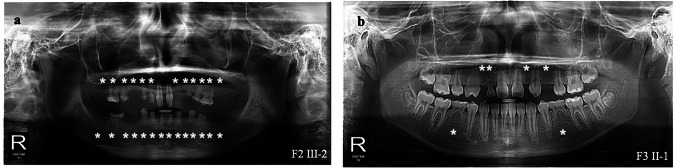
Panoramic radiographs of the two participants in the Family 2 and Family 3. **a** F2 III-2 15 years old girl is missing 26 permanent teeth, conically shaped maxillary incisors, and primary teeth still remain. **b** F3 II-1 15 years old girl is missing 6 permanent teeth and 52, 75, and 85 still remained

Family 1 consisted of a 30-year-old mother and a 12-year-old son as participants, with the mother (F1 II-2) missing nine permanent teeth based on clinical and panoramic radiographs. The middle son (F1 III-2) was also found to be missing eight permanent teeth. Family 2 included a 69-year-old grandmother, a 42-year-old son, and a 15-year-old granddaughter as participants, where the grandmother (F2 I-2) had a total of five missing permanent teeth, her son (F2 II-1) was missing three permanent teeth, and her granddaughter (F2 III-2) was missing a significant number of teeth, specifically twenty-six permanent teeth (Fig. 2a). In Family 3, the participants were a 46-year-old father and his 15-year-old dizygotic twin daughters. The father (F3 I-1) was missing two permanent teeth, and one twin daughter (F3 II-1) missing six permanent teeth (Fig. 2b) while the other twin (F3 II-2) was missing four permanent teeth. The remaining six families did not exhibit inherited patterns, and none of the family members of the participants showed any signs of TA. Family 4 included an 8-year-old girl (F4 II-1) was found to be missing seven permanent teeth, while in Family 5, an 11-year-old male (F5 II-7) was missing six permanent teeth. Family 6 had a 12-year-old boy (F6 II-1) who was missing twelve permanent teeth and in Family 7, an 18-year-old girl (F7 II-1) was missing seven permanent teeth. Family 8 had an 8-year-old girl (F8 II-3) missing six permanent teeth; in Family 9, a 13-year-old boy (F9 II-1) was diagnosed with congenital agenesis and was missing a total of eleven permanent teeth. The participants who exhibit missing teeth, as indicated in the chart, are listed in Table 1. Furthermore, the panoramic radiographs of the remaining participants are presented in Supplement Table 5.

**Table 1:** Phenotype (missing teeth) and genotypes of 5 variants from segregation analysis in 14 participants; minor risk allele in segregation analysis

Family	Patients	Sex	TA phenotype	Number of missing teeth	MAST4-C	ITGA-G	PITX2-G	CACNA1S-G	CDON-G
1	II-2	Female	Affected	9	**C/CGCT**	**G/GA**	**G/A**	G/G	**G/A**
1	III-2	Male	Affected	8	**C/CGCT**	**G/GA**	**G/A**	G/G	G/G
2	I-2	Female	Affected	5	C/C	G/G	**G/A**	G/G	G/G
2	II-1	Male	Affected	3	**C/CGCT**	G/G	**G/A**	G/G	**G/A**
2	III-2	Female	Affected	26	**C/CGCT**	G/G	**G/A**	G/G	**G/A**
3	I-1	Male	Affected	2	C/C	G/G	G/G	**G/A**	G/G
3	II-1	Female	Affected	6	**C/CGCT**	G/G	**G/A**	**G/A**	G/G
3	II-2	Female	Affected	4	**C/CGCT**	G/G	**G/A**	**G/A**	G/G
4	II-1	Female	Affected	7	C/C	G/G	**A/A**	G/G	**G/A**
5	II-7	Male	Affected	6	C/C	G/G	G/G	G/G	G/G
6	II-1	Male	Affected	12	C/C	G/G	G/G	G/G	G/G
7	II-1	Female	Affected	7	C/C	G/G	**G/A**	G/G	G/G
8	II-3	Female	Affected	6	C/C	G/G	**A/A**	G/G	**A/A**
9	II-1	Male	Affected	11	C/C	G/G	G/G	G/G	**G/A**

### Candidate variants identified in WES analysis

WES was performed on all 14 participants who provided their consent. A total of five variants that met all the filtering criteria were identified through segregation analysis. Participants from inherited families with non-syndromic TA demonstrated autosomal dominant inheritance patterns, while those non-inherited families exhibited autosomal recessive inheritance (Table 1 and 2). Among the Asian population, two rare missense variants were found: rs3850625 in CASNA1S with an Asian MAF of 0.0419 and rs12274923 in CDON with an Asian MAF of 0.0629. Additionally, a disruptive in-frame insertion in MAST4 (rs201910335) with an Asian MAF of 0.014 was also identified.

**Table 2:** A record of filtered variants. Gene, gene name; ID, variant id in SNP; CHR, chromosome; POS, base pair position; REF, reference allele; ALT, alternative allele

Gene	ID	CHR	POS	REF	ALT	Transcript ID	Protein change	Impact
MAST4	rs201910335	5	65892764	C	CGCT	ENST00000404260	Leu95dup	MODERATE
ITGA6	-	2	173337540	G	GA	ENST00000409532	Asp114fs	HIGH
PITX2	rs2278782	4	111542154	G	A	ENST00000557119	Gln193*	HIGH
CACNA1S	rs3850625	1	201016296	G	A	ENST00000367338	Arg1520Cys	MODERATE
CDON	rs12274923	11	125871715	G	A	ENST00000531738	Ala63Val	MODERATE

### In silico mutation impact analysis of the variants

**Fig. 3:**
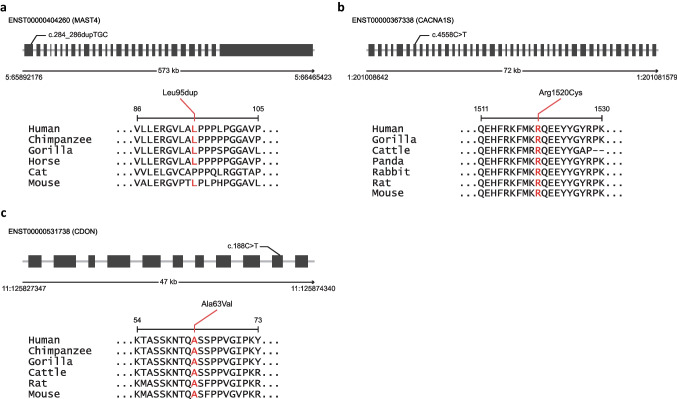
Mutation impact analysis of MAST4, CACNA1S, and CDON **a** The amino acid sequence alignment of MAST4 in various mammalian orthologs revealed a mutated residue of Leu95dup, which is highlighted in red. **b** The amino acid sequence alignment of CACNA1S in various mammalian orthologs revealed a mutated residue of Arg1520Cys, which is highlighted in red. **c** The amino acid sequence alignment of CDON in various mammalian orthologs revealed a mutated residue of Ala63Val, which is highlighted in red

Two different computational tools were applied to analyze the impact of mutations in MAST4, CACNA1S, and CDON with filtered variants: SIFT [47] and PolyPhen2 [48]. For the two missense variants, canonical Ensembl sequences were analyzed (Table 3). The SIFT predicted a damaging effect on a missense variant in CACNA1S, while PolyPhen2 did not. In contrast, a variant in CDON was predicted to be possibly damaging by PolyPhen2 and had both damaging and tolerant prediction by SIFT. The variants were found to be located in evolutionarily conserved positions and might be under intense selective pressure. For instance, a missense variant in CACNA1S (located in exon 7, and arginine at the amino acid position 1520) and a variant in CDON (located in exon 10, and alanine at the amino acid position 63) were well-conserved in mammal orthologs (Fig. 3b, c). In the case of MAST4, the mutation has occurred at the site where sequences are well-conserved across mammal orthologs (Fig. 3a). To assess the gene tolerance to mutations concerning TA, we measured Loss-of-Function observed/expected upper bound fraction (LOEUF) scores. A lower LOEUF score indicates that a gene is more susceptible to damage by mutations and may have a more severe impact on affected individuals [49]. We found that MAST4 had a relatively low LOEUF score of 0.38 and that other genes also had LOEUF scores below 1(CACNA1S had a LOEUF score of 0.56 and CDON had a LOEUF score of 0.96), suggesting that the gene functions might be damaged by the variants (Table 3).

**Table 3:** Analysis of mutation impact on candidate genes; results of PolyPhen2 (B, benign; P, possibly damage) and SIFT (D, damage; T, tolerate). Effect, defining mutations; LOEUF, indicates the inverse score of loss-of-function intolerance; ASN MAF, alternative allele frequency in 1000Gp1 Asian descendent samples; MAF, alternative allele frequency in whole 1000Gp1 data

Gene	UniProt ID	Effect	PolyPhen2	SIFT	LOEUF	MAF	ASN MAF
MAST4	O15021	disruptive_inframe_insertion	-	-	0.38	0.005814	0.01434
CACNA1S	Q13698	missense_variant	B	D, D	0.56	0.070971	0.041958
CDON	Q4KMG0	missense_variant	P, P, P	T, D, T	0.96	0.125916	0.062937

### Gene set enrichment analysis

**Fig. 4:**
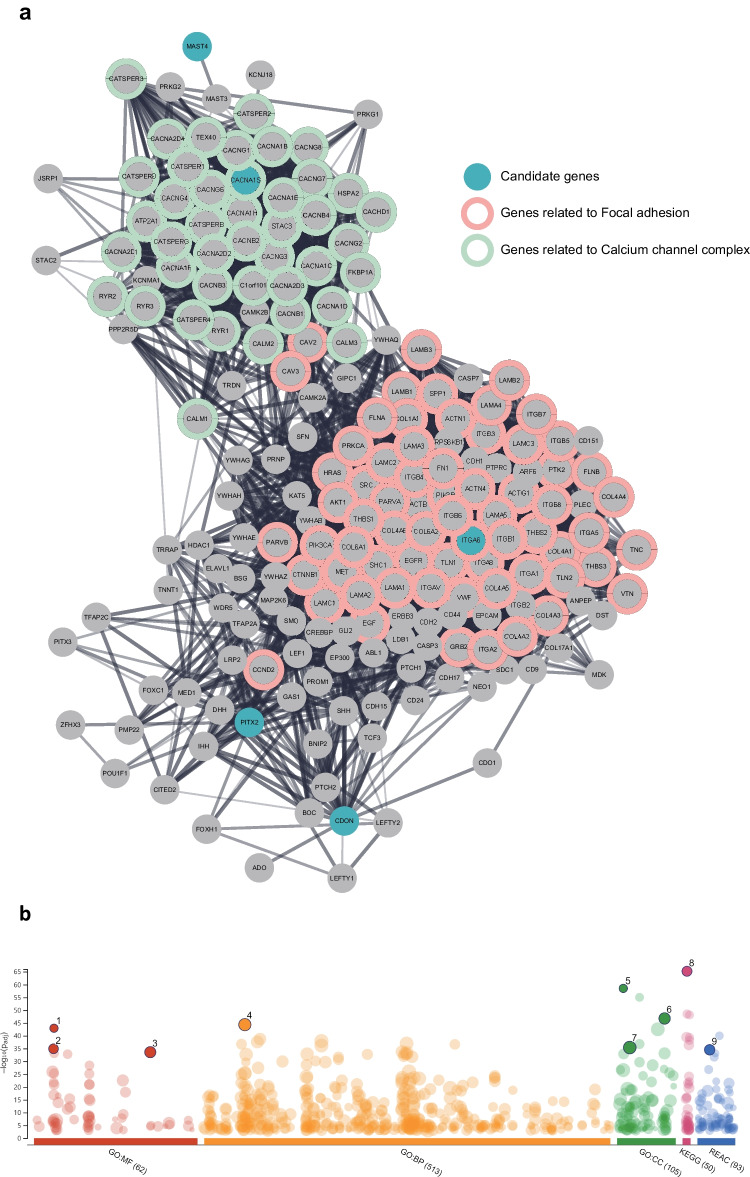
Gene set enrichment analysis result of candidate genes and its PPI network neighboring genes. **a** The PPI network of expanded gene set. Cyan-colored nodes are the five candidate genes. **b** Adjusted *p*-values of function terms as the result of GSEA. Highlighted dots are the high-ranked function terms related to the focal adhesion and calcium channel complex

Next, to comprehend the functional contribution of candidate genes in non-syndromic TA, an investigation of gene set enrichment analysis (GSEA) was performed (Table 4 and Supplement Table 2). This analysis uncovered three noteworthy categories, namely Gene Ontology (GO), Kyoto Encyclopedia of Genes and Genomes (KEGG), and Reactome pathway (REAC). For the GSEA, we expanded the list of genes with neighbors in the PPI network. Using the five candidate variant genes, function terms related to focal adhesion and calcium channel complex were highly ranked and formed functional gene clusters in the PPI network (Fig. 4).

**Table 4:** List of highlighted function terms of the gene set enrichment analysis result plot

	Source	Term	Term ID	Count	*p*-value
1	GO:MF	voltage-gated calcium channel activity	GO:0005245	28	1.10E-43
2	GO:MF	integrin binding	GO:0005178	35	1.23E-35
3	GO:MF	cell adhesion molecule binding	GO:0050839	52	2.66E-34
4	GO:BP	cell adhesion	GO:0007155	87	4.42E-45
5	GO:CC	voltage-gated calcium channel complex	GO:0005891	34	2.80E-59
6	GO:CC	plasma membrane protein complex	GO:0098797	66	1.69E-47
7	GO:CC	cell junction	GO:0030054	87	4.01E-36
8	KEGG	Focal adhesion	KEGG:04510	67	6.90E-66
9	REAC	Extracellular matrix organization	REAC: R-HSA-1474244	50	3.11E-35

## Discussion

To the best of our knowledge, this is the first study to use a combination of WES and bioinformatic analysis to comprehensively investigate the genetic factors underlying functional mechanisms associated with TA in Mongolian families. TA is a congenital disorder characterized by the absence of one or more teeth due to the failure to develop. The underlying mechanism of TA is complex and still needs to be fully understood [14]. In recent years, there has been a growing interest in utilizing WES and bioinformatic analysis to identify pathogenic variants in candidate genes associated with TA [30]. WES is a highly effective method for identifying genetic variants associated with genetic diseases, and genes involved in the developmental and differentiation of tooth germ cells have been discovered using this method [28].

Although new variants are still being discovered, the exact pathogenic mechanism underlying TA remains unclear. Previous studies on TA have primarily focused on genetic variants, with a limited investigation into the functional aspects such as protein function or molecular pathways. In this study, we aim to achieve an understanding of TA by investigating not only the genetic variants but also the underlying functional mechanisms involved in the process. To contribute to this, we conducted a bioinformatic analysis that included a comparison of the identified genetic variants with public databases and literature, as well as an assessment of the frequency of the variant in the general population (LOEUF analysis), and a prediction of the potential impact of the variant on protein function (SIFT and PolyPhen analyses).

In our study, we found impact variants in MAST4. The result of variant impact analysis in the general population revealed that MAST4 showed a lower LOEUF score of 0.38 (Table 3) compared to CACNA1S and CDON, suggesting it could be related to the tooth damage and have a more severe impact on affected individuals. Indeed, a disruptive variant (rs201910335) in the MAST4 gene was identified in 6 individuals from three inherited families with TA (Table 1). The MAST4 gene was first characterized in 2006 through bioinformatics analysis [50]. Studies have investigated its roles in crucial biological processes, including cell cycle progression, cell migration, and neuronal development [51]. The precise mechanism by which the MAST4 gene leads to TA is not fully understood up to this point. However, a recent study suggests that MAST4 is closely involved in the amelogenesis process of mouse incisors and may serve as a critical regulator of Amelogenesis Imperfecta [52] by deactivating Wnt/$\beta$-catenin signaling pathway [53]. This result is particularly noteworthy and captures our attention because the Wnt signaling pathway is also a major pathway in TA [54, 55]. During tooth formation, the Wnt signaling pathway is activated in the dental epithelium, which leads to the formation of a structure called the enamel knot. The enamel knot serves as a signaling center that helps direct the dental mesenchyme’s growth and differentiation [55]. Mutations or alterations in the genes involved in the Wnt signaling pathway, such as WNT10A, AXIN2, and LRP6, are known to be associated with TA. Mutations in WNT10A gene, which encodes a ligand for the canonical Wnt pathway, have been linked to TA [56]. The mutations in AXIN2, which is a negative regulator of the gene canonical Wnt pathway, have also been associated with TA, as well as other dental abnormalities [57]. In addition, variations in the LRP6 gene, which encodes a co-receptor for the canonical Wnt pathway, have been linked to TA [58]. These findings highlight the significance of Wnt and Wnt-associated pathways in the genetic etiology of TA and may facilitate the identification of novel genes associated with this condition. Our results suggest that the MAST4 gene’s relationship with the Wnt pathway may significantly contribute to the development of TA. However, further research is necessary to comprehensively elucidate the underlying molecular mechanism through which the MAST4 and related pathways influence tooth development and contribute to the pathogenesis of TA.

We identified a stop-gained mutation in PITX2 gene (rs2278782) in 8 participants from inherited and non-inherited families (Table 1). This finding is consistent with previous research linking PITX2 mutation to non-syndromic TA and dental anomalies [32, 59]. PITX2 is a transcription factor for proper tooth development by activating target genes through the Wnt signaling pathway, involving $\beta$-catenin [60]. Mutation on the PITX2 gene can disrupt the proper secretion of Wnt4, Wnt6, and Wnt10 from dental epithelium, resulting in a dysfunctional enamel knot leading to the arrest of tooth development due to the absence of Wnt/$\beta$-catenin activity [61–63]. Variants in CDON and ITGA6 genes were also found, which are co-receptors for the SHH signaling pathway crucial for tooth development [64, 65]. The SHH signaling affects cell polarization in the early tooth bud, determining the number of teeth in the permanent dentition. It also interacts with other pathways, including Wnt, to ensure proper tooth development [66]. Therefore, our findings suggest that the Wnt signaling pathway leads to TA.

Gene set enrichment analysis revealed the functional terms of five candidate genes associated with focal adhesion and calcium channel complex to be highly important, with two gene clusters linked by the candidate genes and their neighboring genes in the PPI network. Focal adhesions are specialized structures that allow cells to interact with extracellular matrix (ECM) through multiprotein complexes, mediating cell adhesion, migration, and signaling processes [67, 68] necessary for the formation of the tooth germ, enamel, dentin, and periodontal ligament [69–71]. Inhibiting focal adhesion kinase (FAK) can hinder the bud-to-cap morphogenesis process, potentially leading to TA [72, 73]. Calcium signaling, regulated by voltage-gated channels (VDCC) [74] and important in tooth development [75], can be disrupted by mutations in calcium channel genes, such as CACNA1S, leading to dental malformations and abnormalities [76]. These findings indicate that disruptions in focal adhesion may affect tooth morphogenesis and differentiation, which may lead to TA. Furthermore, the significance of calcium signaling in tooth development suggests that calcium channelopathies can contribute to dental abnormalities.

In light of the limitation of our study, specifically the inclusion of participants with varying degrees of TA in the same analysis, it would be beneficial for future investigations to stratify participants into distinct subgroups based on the severity of TA. This stratification would allow for a more refined analysis and deeper understanding of the specific mutation types and genetic factors associated with each form of TA. Additionally, the relatively small sample size in our study may have limited the statistical power to detect smaller effects or associations accurately. While we have made efforts to address these limitations through bioinformatic analysis and functional analysis, larger and more homogeneous cohorts are needed to further validate and expand upon our findings. Nonetheless, despite these limitations, our study provides valuable insights into the genetic basis of TA and identifies potential candidate genes associated with this condition.

In summary, our study has identified novel candidate genes and variants that contribute to the occurrence of non-syndromic TA in Mongolian families. Furthermore, our results emphasize the significance of Wnt pathway genes, as well as focal adhesion and calcium channel complexes, in regulating the process of tooth development. Future research aimed at exploring the specific functions of genes and complexes, both individually and in combination, could provide a better understanding of the etiology of TA. Additionally, our results may guide clinical practice, inform clinical gene diagnosis, and facilitate the development of targeted treatment options for individuals affected by TA.

## Supplementary Information

Below is the link to the electronic supplementary material.

Supplementary Fig. 1 (PNG 122 kb)

High resolution image (EPS 1654 kb)

Supplementary Tables (XLSX 827077 KB)

## Data Availability

The datasets utilized and examined in the present study can be obtained from the corresponding author upon a reasonable request.
