# Supplementary figures and images for: Identification of novel candidate genes associated with non-syndromic tooth agenesis in Mongolian families
Source: Clin Oral Investig. 2023 Dec 29;28(1):56. doi: 10.1007/s00784-023-05415-2 (PMC10756872; doi:10.1007/s00784-023-05415-2)

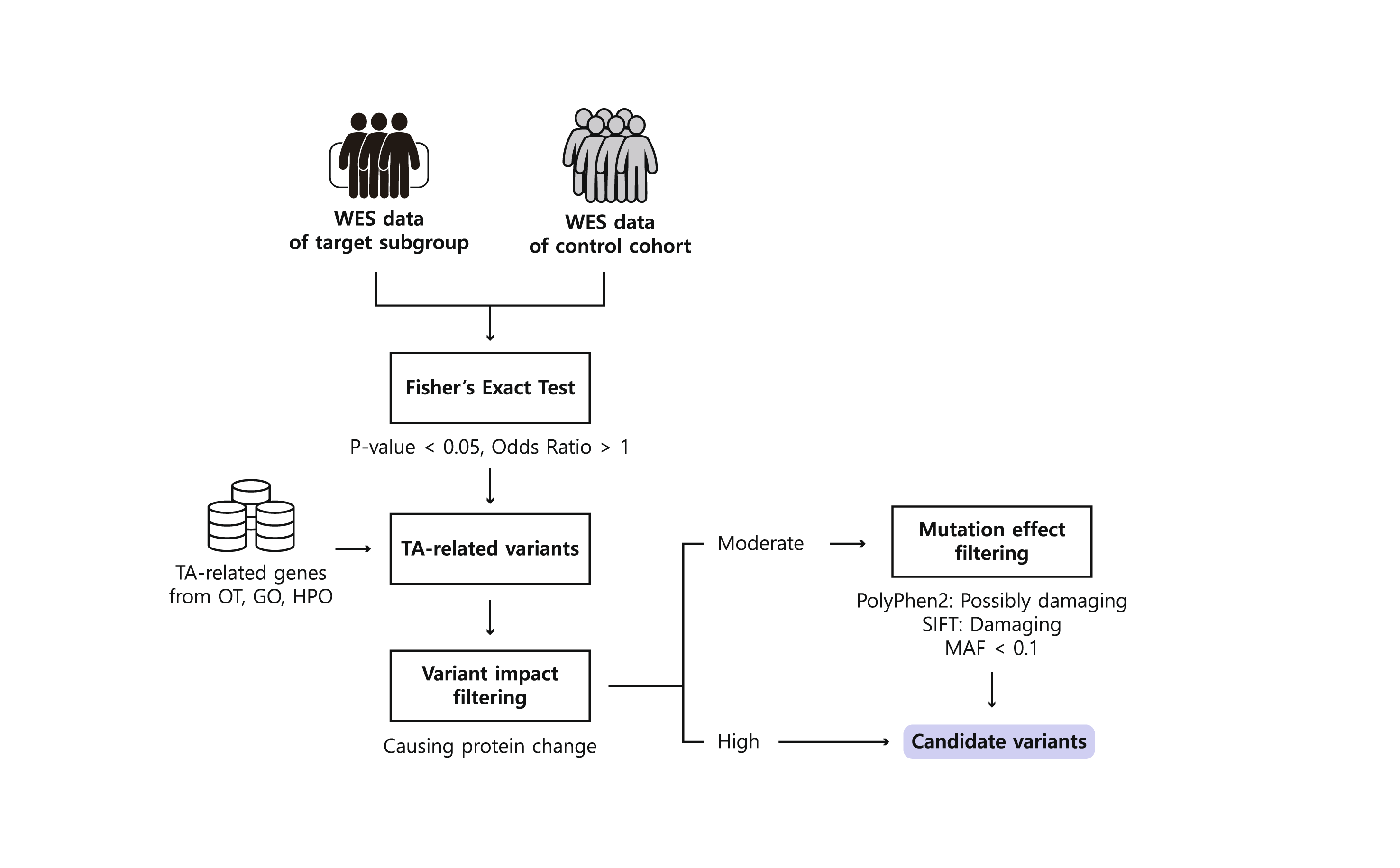

Supplement: Supplementary file 1 — (PNG 122 kb) [file 784_2023_5415_Fig5_ESM.png]
